# Brown midrib (BMR) and plant age impact fall armyworm (*Spodoptera frugiperda*) growth and development in sorghum-sudangrass (*Sorghum x drummondii*)

**DOI:** 10.1038/s41598-024-63397-x

**Published:** 2024-06-02

**Authors:** Alejandro Vasquez, Devi Balakrishnan, Jessica Ayala, Kelly Loftin, Joe Louis, Rupesh Kariyat

**Affiliations:** 1 Department of Entomology and Plant Pathology, University of Arkansas, Fayetteville, AR 72701 USA; 2 Department of Entomology, University of Nebraska-Lincoln, Lincoln, NE USA; 3 Department of Biochemistry, University of Nebraska-Lincoln, Lincoln, NE USA

**Keywords:** Insect-plant interactions, Pest management, Herbivory, Fall armyworm, Brown midrib, Larval performance, Agroecology, Ecosystem services

## Abstract

Economic losses from insect herbivory in agroecosystems has driven the development of integrated pest management strategies that reduce pest incidence and damage; however, traditional chemicals-based control is either being complemented or substituted with sustainable and integrated methods. Major sustainable pest management strategies revolve around improving host plant resistance, and one of these traits of interest is Brown midrib (BMR). Originally developed to increase nutritional value and ease of digestion for animal agriculture, BMR is a recessive plant gene usually found in annual grasses, including sorghum and sorghum-sudangrass hybrids. In sorghum-sudangrass, BMR expressed plants have lower amounts of lignin, which produces a less fibrous, more digestible crop, with possible implications for plant defense against herbivores- an area currently unexplored. Fall Armyworm (FAW; *Spodoptera frugiperda*) is a ruinous pest posing immense threat for sorghum producers by severely defoliating crops and being present in every plant stage. Using FAW, we tested the effect of seed treatment, BMR, and plant age on FAW growth, development, and plant defense responses in sorghum-sudangrass. Our results show that seed treatment did not affect growth or development, or herbivory. However, presence of BMR significantly reduced pupal mass relative to its non-BMR counterpart, alongside a significant reduction in adult mass. We also found that plant age was a major factor as FAW gained significantly less mass, had longer pupation times, and had lower pupal mass on the oldest plant stage explored, 60-days, compared to younger plants. These findings collectively show that pest management strategies should consider plant age, and that the effects of BMR on plant defenses should also be studied.

## Introduction

Sorghum (*Sorghum bicolor*), is one of the most agriculturally important crops grown across the world, historically being grown for centuries in Africa and Asia^1^.However, sorghum production in the United States has exponentially increased since the 1980s, and the United States is now producing approximately 25% of the world’s sorghum crop^1,2^. Similar to other grasses, sorghum and sorghum-sudangrass (*Sorghum x drummondii*) face attack from over 150 insect species, many of them attacking specific parts of the plant that include, but are not limited to, sorghum shoot fly (*Atherigona soccata*), multiple stem borers (*Chilo partellus*, *Busseola fusca*, *Diatraea saccharalis*), armyworms (*Spodoptera exemtpa* and *S. frugiperda*), aphids (*Melanaphis sacchari*, *Schizaphis graminum*), amongst others^3,4^. To combat the large swath of pests that constantly damage sorghum leading to yield loss, growers employ various management strategies not only based on target pests, but also reliant on cost and location. For example, in North America *Lepidopteran* pests have historically been managed in sorghum, maize, and similar crops primarily through transgenic hybrid crops expressing the insecticidal properties of *Bt* (*Bacillus thuringiensis*), or genetically altered crops to produce elevated levels of defense compounds, one example being flavonoids^5,6^.Conversely, in Asian and African agricultural systems with smaller holding sizes, cultural practices involving early planting and intercropping are the primary methods utilized for the management of sorghum pests^6,7^.

While cultural practices are well incorporated in many regions, they have not been found to be the most impactful in reducing yield loss due to herbivory^8^ and similarly *Bt* crops have shown accelerated *Bt* resistance in insect populations from fields without lengthy exposure to use of *Bt* crops^9,10^. This unfilled gap in easily applied sorghum-pest control, paired with the increase in sorghum production in the United States warrants new integrated pest control management strategies be developed. Alongside this rapid increase in sorghum production comes a rapid increase of sorghum pests the region is not accustomed to managing- and fall armyworm, FAW (*Spodoptera frugiperda*)^11^ is a major one*.*

FAW is a widely distributed, polyphagous generalist herbivore pest known primarily for its destructive defoliation during larval stages^12–14^. Despite its destructive notoriety, FAW appears to be recently emerging in regions unable to manage them such as Egypt^15^, Uganda^16^, and India^17–19^, and it is forecasted to extend more as global climate patterns change^20^. Additionally, since *Bt* and cultural practices may not achieve the desired level of FAW control, research has shifted toward harsher insecticide practices and in particular, seed treatments^21,22^. While Muraro’s group^22^ found a reduction in FAW damage on seed treated maize plants 7 and 14 days after emergence, after 20 days seed treated maize showed no difference in FAW damage when compared to maize without seed treatments. Chanda’s group^21^ also observed a delayed infestation via seed treatment coatings in maize; however, failed to find any decrease in FAW damage after infestation. Another study^23^ evaluated the effect of seed treatments on FAW via laboratory bioassays of soybean seed treatments and found significant reductions in FAW survival in most treatment evaluations within key growth stages. Clearly, there is an urgent need to explore alternative methods to manage FAW, including a closer look at host plant resistance.

Brown midrib (BMR) is a recessive plant trait that is visually seen as a reddish-brown pigmentation in the leaf midrib and stem of the plant, usually in sorghum, maize, and millets^24^. The reddish-brown pigmentation has largely been found to be associated with lower lignin levels, alongside an assumed increase of its digestibility as forage^24–27^. For example, studies have found BMR plants lower in neutral detergent fiber (NDF) and acid detergent lignin (ADL) concentrations when compared to non-BMR genotypes^28^. The increased digestibility because of lower lignin content has implications for animal agriculture, as dairy cow milk yield, milk protein, and milk fat content was not affected by a BMR diet, which is lower in cost to the producer, when compared to a traditional corn silage diet^29^. In integrated pest management, BMR has been found to have differential effects based on the type of insect pest being controlled, as a result of the genetic changes to the plant from the BMR trait^30–32^. For example, Dowd^31^ found Lepidopteran pests (*H. zea, S. frugiperda*) weighed significantly less when feeding on BMR leaves than those feeding on wild-type leaves, and mortality showed similar trend. Much of the implications of BMR as it relates to insects revolves around pest species, primarily herbivores, but involves resistance against microorganisms and diseases as well^33–35^. Because of its increased digestibility, it has been thought that BMR plants are generally more susceptible to insect herbivory. However, a study shows a neutral result when looking at digestibility as a factor, showing that final instar Southern armyworm (*Persectania ewingii*) that were fed seedling *Triticum aestivum* with differing amounts of NDF, neutral detergent solubles, and dry matter, did not lead to increased mass gain in the caterpillars regardless of higher digestibility, nor was there a difference in overall consumption^33^. Another group, while they found no difference in consumption between BMR and non-BMR plants, found BMR plants had significantly higher density of thrips, corn leaf aphids (*Rhopalosiphum maidis*), and were also more susceptible to FAW^34^. Collectively, the results from these studies warrant additional examination of BMR on insect herbivory.

While higher in digestibility, there are concerns that reduced lignin makes BMR mutant plants more vulnerable to stalk rot and weaker in stalk strength. Similar to caterpillars not gaining increased mass on BMR plants, Tesso and Ejeta^35^ found BMR plants did not exhibit stalk collapse as a result of proposed lower stalk strength, nor were they more susceptible to stalk rot after *Macrophomina phaseolina* inoculation. When comparing BMR sorghum to non-BMR sorghum, it was shown that BMR sorghum tissues to be more readily degraded than similar tissues in the non-BMR line^36^. This was due to BMR plant leaf blades having significantly lower NDF, blades and sheaths lower in permanganate lignin (PML), and tissues higher in in vitro dry matter digestibility^36^. Mutant BMR plants showed higher levels of aldehydes, alongside increased presence of benzodioxane when compared to wild type leaves^37^. BMR plants have also been found to have lower trans-p-coumaric acid concentrations, and a lower p-coumaric acid to ferulic acid ratio^28^. Coumaric acid is known to be used by plants to induce defense strategies, primarily against insects^38^. Coumaric acid directly deterred both *S. litura* and *Amsacta albistriga* via antiherbivore effects, while indirectly deterring both herbivores via natural enemy attraction, the parasitoid *Trichogramma chilonis*^39^. Similar antibiosis effects of coumaric acid have been observed in other lepidopterans, such as *Chilo partellus*^40^. The mechanism of its anti-herbivory properties has been explained by Lim^41^, which found coumaric acid inhibiting tyrosinase, a known key enzyme for the insect molting process. Since most studies on BMR and pest management shows differential and species-specific effects, our goal was to critically examine how the differences between BMR and non-BMR sorghum affects FAW life history traits, and whether they are affected by host plant age.

To examine the role of BMR, plant age, and in host plant resistance of sorghum-sudangrass hybrids against FAW, we used a combination of greenhouse and lab experiments utilizing three sorghum-sudangrass genotypes: two BMR and one non-BMR genotype each with a seed treatment made up of two fungicides, fludioxonil and metalaxyl, and our insecticide being evaluated, spinosad, treated by and obtained from Richardson Seeds (Vega, Texas, USA) alongside non-treated control seeds. We used sorghum-sudangrass as our study system since the crop is commonly grown for feed, has been previously used for similar studies^42^, and was commercially available with BMR and non-BMR genotypes. We then designed FAW growth and development bioassays structured around the following questions: (1) does the presence of BMR influence the growth and development of FAW under short exposure (2) does the presence of BMR influence the amount of herbivory on sorghum-sudangrass plants at different plant ages, (3) do commercially available seed treatments influence the growth and development of FAW, and (4) whether FAW exposed to BMR sorghum-sudangrass or seed-treated sorghum-sudangrass are able to pupate and eclose successfully, measured in pupal and adult mass? We hypothesized that BMR will have differential effects on the growth and development of FAW on BMR sorghum-sudangrass but also on herbivory related injury suffered by the plant. We predicted that older sorghum-sudangrass plants will be more detrimental to FAW development, based on a previous study that reported more damage on younger BMR leaves and significantly less damage on older BMR leaves^31^. We also hypothesized that seed-treated sorghum-sudangrass will have little to no effect on the growth and development of FAW, nor will it reduce its herbivory as multiple studies have shown low success of systemic insecticides, especially in later instars^43,44^.

## Materials and methods

### Plant material-sorghum-sudangrass

For all sorghum-sudangrass plant genotypes (S425 - No BMR) treated and untreated, S60 (Contains BMR) treated and untreated, S72 (Contains BMR) treated and untreated, described in this study were obtained from Richardson Seeds (Richardson Seeds Ltd, Vega, Texas, USA). These genotypes were chosen because of their similarities in growth traits. Plants were grown in DL33 Deepot Tree Pots (6.9 cm diameter, 20.3 cm deep, Greenhouse Megastore, Danville, Illinois, USA) with seeds being sown in LB15 potting soil (Farmers Co-Op, Van Buren, Arkansas, USA). The plants were fertilized with Osmocote Plus 15-9-12 (ICL Specialty Fertilizers) every 14 days and received iron chelate micronutrient (Sprint 330 Chelated Iron 10%) every 14 days. Plants were grown in a greenhouse with a 16-h-light/8-h-dark photoperiod, 28 °C, 50–60% relative humidity. The following experiments were replicated on all three of the following sorghum-sudangrass stages, indicated in days after germination: 10-days old (3-leaf stage), 25-days old (panicle inflation stage), 60-days old (booting stage). Voucher specimens for the species have been previously deposited (after identification) at herbarium from previous work on this species. Permissions to conduct experiments and collect seeds have been obtained from the seed company. All experimental protocols followed institutional, national, and international guidelines and legislation.

### Insects- fall armyworm

FAW were purchased as eggs (Frontier Agricultural, Newark, Delaware, USA). FAW eggs were allowed to hatch inside the laboratory at 25 °C and were then reared on an artificial wheat-based germ diet (Product Code: F9772; Frontier Agricultural, Newark, Delaware, USA). The diet was made as per specifications from the supplier, as well as our previous work: 1000 mL of water was heated in an iron cooking pot on a hot plate with mechanical stirring until boiling, followed by the addition of 200 g of General-Purpose Lepidoptera Diet added in slowly to be thoroughly mixed without clumping. Once thoroughly mixed, 8 g Agar powder was added into the mixture and mixed thoroughly again. The completed mixture was added to plastic Sterilite 6-quart storage boxes (Walmart; Bentonville, Arkansas, USA) and left at room temperature for 4 hours for cooling before being refrigerated^42^.

### Experiments

#### Snapshot exposure experiment

FAW were reared on artificial diet until reaching 2nd instar, at which larvae were then weighed before being placed on sorghum-sudangrass plants in the greenhouse. FAW were placed within bags, and these bags were then securely tied to sorghum-sudangrass plants using drawstring organza bags (10.2 cm × 15.2 cm, Volcanic, Amazon, Seattle, Washington, USA) with one FAW per plant. Fifteen caterpillars were individually placed on each plant per each of the 6 treatments, S425 (non-BMR) treated and untreated, S60 (BMR) treated and untreated, S72 (BMR) treated and untreated (N = 90), alongside 30 caterpillars continuing to be reared on artificial diet as a control (N = 30). FAW fed on plant material within the bag for 48 h before being removed from the plant and weighed again. Plant tissue fed on by FAW was then scaled from 0–4 to analyze extent of herbivory on each individual plant^45^. Pre-exposure and post-exposure mass were then used to calculate mass gain normalized for initial mass^42^. This experiment was replicated on all three of the stated sorghum-sudangrass stages, 10-days old (3-leaf stage), 25-days old (panicle inflation stage), 60-days old (booting stage).

#### Continuous FAW exposure experiment with fresh leaves

For this experiment, FAW eggs were allowed to hatch inside the laboratory at 25 ℃ and were then reared exclusively on freshly cut sorghum-sudangrass leaves (leaves harvested for these bioassays were from young, fully developed from the top, ~10 leaves) from each of the six treatments: S425 (non-BMR) treated and untreated, S60 (BMR) treated and untreated, S72 (BMR) treated and untreated, N = 120. FAW were individually placed in plastic cups closed with lids (Dart Container Corporation, Mason, Michigan, USA) with enough plant material to never be starved. Cups were cleaned daily to avoid excess humidity and frass, alongside sorghum-sudangrass leaves replaced daily. Twenty caterpillars were reared on an artificial wheat-based germ diet, as described previously, to serve as control, N = 20. Once caterpillars reached 2nd instar, they were weighed daily for analysis of growth and development^46^. This experiment was also replicated on all three stages, 10-days old (3-leaf stage), 25-days old (panicle inflation stage), 60-days old (booting stage).

#### Plant-based diet exposure experiment

In this experiment, FAW eggs were allowed to hatch inside the laboratory at 25 ℃ and were then reared on different artificial wheat-germ-based diets. Control diet was made following supplier specifications of 1000 mL water, 8 g agar powder, and 200 g Lepidopteran diet powder; however, plant-based diets were made with the addition of 10% plant material (plant material was selected from the top of the plant, taking multiple, large fully developed leaves), such as 20 g plant material: 180 g Lepidopteran diet powder, based on our previous studies^42^ to test host plant toxicity under controlled conditions. For this experiment, freshly collected leaf material was finely ground using mortar-and-pestle, and then added to the diet just before cooling. FAW were then placed prior to hatching on plant-based diets from each of the 6 treatments, S425 (non-BMR) treated and untreated, S60 (BMR) treated and untreated, S72 (BMR) treated and untreated, and control, N = 140, in the similar plastic cups as mentioned before. Artificial diet was cut into ~1 cm^3^ blocks and replaced every 2 days to avoid desiccation and excessive moisture build up in the cup. Once caterpillars reached 2nd instar, they were weighed daily for estimating growth and development. This experiment was also replicated with plant material from all three sorghum-sudangrass stages, 10-days old (3-leaf stage), 25-days old (Panicle Inflation Stage), 60-days old (Booting Stage).

### Statistical analysis

For all the experiments, our statistical model had four factors: sorghum-sudangrass age (10d, 25d, 60d), sorghum-sudangrass genotype (S425, S60, S72) seed treatment (untreated, treated), and BMR presence (No BMR, contains BMR).We pooled genotypes to narrow our scope to age, seed treatment, and BMR presence, and used Analysis of Variance for continuous variables (Total Mass Gain, Early Mass Gain, Late Mass Gain, Time to Pupation, Pupal Mass, and Adult Mass), and Ordinal logistic regression for discrete scale date (Plant Damage). Pairwise post hoc comparisons were carried out using Tukey’s test.

## Results

### Snapshot exposure experiment

#### Mass gain

Pre- and post-exposure caterpillar weights (g) were recorded and mass gain was then calculated expressed as a %.

$$Mass\,\, Gain (\%)=\left(\frac{\text{FinalMass}-\text{Initial\,\, Mass}}{\text{Initial\,\, Mass}}\right)\times 100.$$

**Figure 1:**
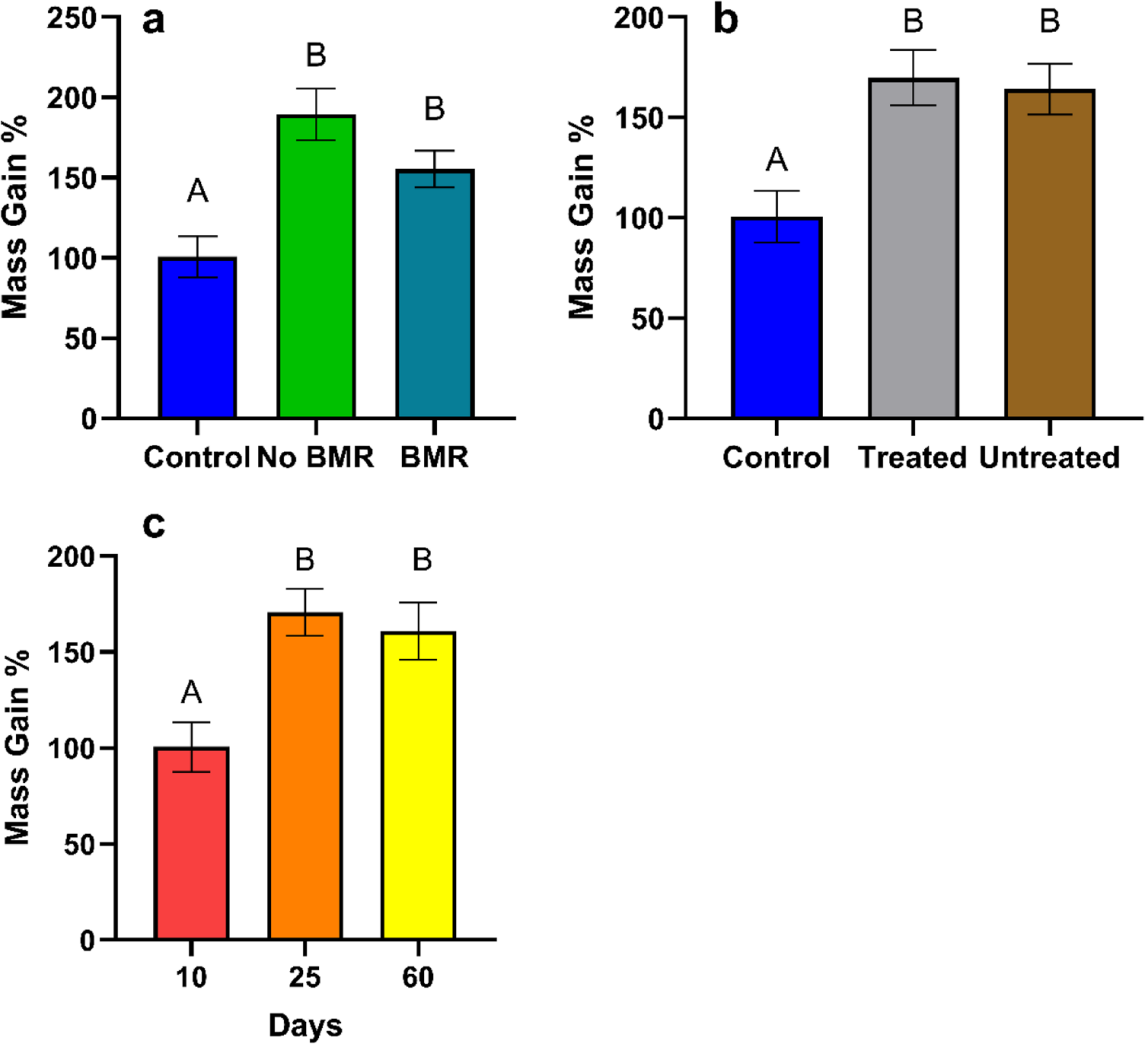
Mean mass gain of 48 h snapshot exposure experiments for caterpillars on (**a**) Artificial diet control, non-BMR, BMR. (**b**) Artificial diet control, seed-treated, no seed treatment (**c**) 10-day old plants, 25 day old plants, and 60-day old plants. Each error bar is constructed using 1 standard error from the mean, different letters above the bars indicate significant differences among total mass gain between BMR treatments, seed treatments, and plant age determined by post hoc analyses using Tukey’s test (P < 0.05).

Mass gain analysis for the effect of BMR (ANOVA: P < 0.0001, Fig. 1a) found no significant difference between BMR (Mean ± SE, 155.3 ± 11.69), and non-BMR (189.4 ± 21.48), yet BMR (P = 0.0047) and non-BMR (P < 0.0001) both FAW gained significantly more mass than the artificial diet control (100.67 ± 8.736). Mass gain analysis for the effect of seed treatment (ANOVA: P = 0.0002, Fig. 1b) found no significant difference between treated (169.8 ± 16.93), and untreated (164.2 ± 13.4), yet treated (P = 0.0009) and untreated (P = 0.0016) both gained significantly more FAW mass than the artificial diet control (100.7 ± 8.734). Mass gain analysis for the effect of plant age (ANOVA: P < 0.0001, Fig. 1c) found caterpillars exposed to 25-day (170.8 ± 13.2; P = 0.0002) and 60-day (166.9 ± 18.2, P = 0.0021) plants gained significantly more mass than caterpillars exposed to 10-day plants (100.3 ± 9.04).

#### Plant damage

**Figure 2:**
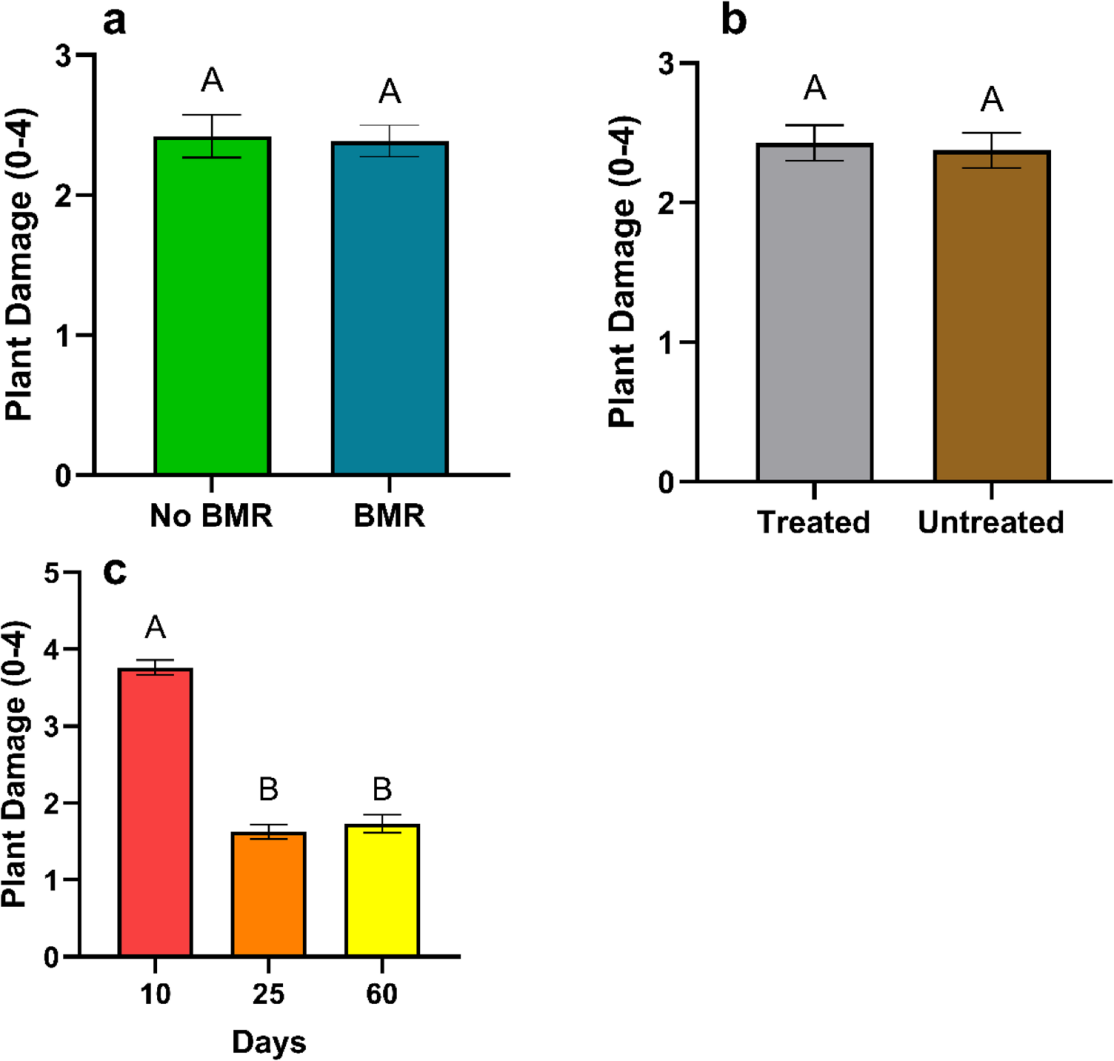
Mean plant damage (0–4) of 48 h snapshot exposure experiments for caterpillars on (**a**) non-BMR and BMR. (**b**) Seed-treated and no seed treatment (**c**) 10-day old plants, 25-day old plants, and 60-day old plants. Each error bar is constructed using 1 standard error from the mean, different letters above the bars indicate significant differences among plant damage between BMR treatments, seed treatments, and plant age determined by post hoc analyses using Tukey’s test (P < 0.05).

Plant damage was measured on a scale (0–4; 0:0%, 1:25%, 2:50%, 3:75%, 4:100%) following the methods described previously^45^. There was no difference in plant damage between BMR plants, and non-BMR plants (ordinal logistic regression (OLR): P = 0.8370, Fig. 2a), and between seed-treated and untreated plants (OLR: P = 0.8718, Fig. 2b); however, 10-day old plants had significantly more caterpillar damage (OLR: P < 0.0001, Fig. 2c) than 25-day and 60-day old plants.

### Continuous fresh leaf exposure experiment

#### Total average mass gain

**Figure 3:**
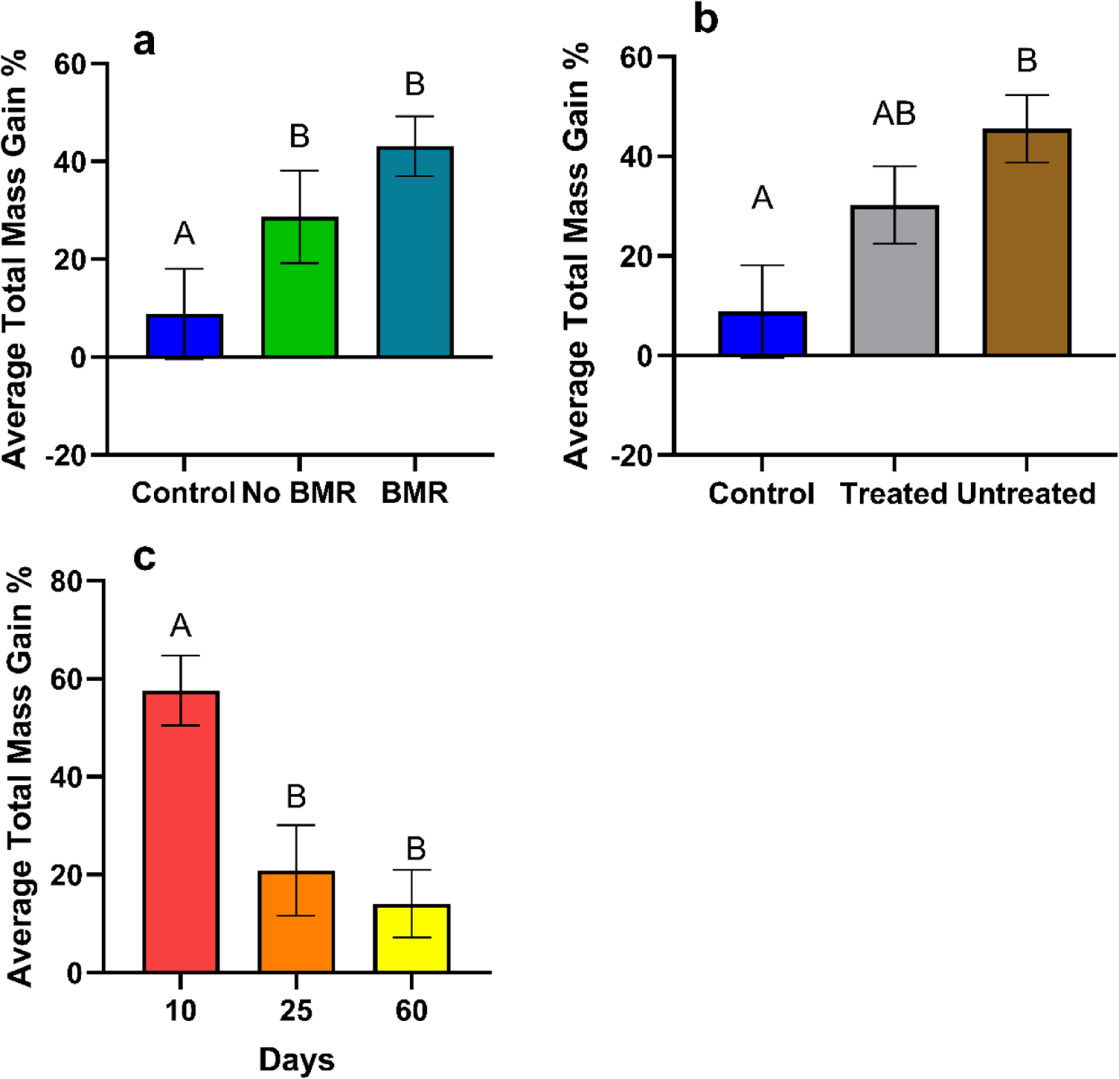
Mean total average mass gain of Continuous leaf exposure experiments for caterpillars on (**a**) Artificial diet control, non-BMR, BMR. (**b**) Artificial diet control, seed-treated, no seed treatment. (**c**) 10-day old plants, 25-day old plants, and 60-day old plants. Each error bar is constructed using 1 standard error from the mean, different letters above the bars indicate significant differences among total mass gain between BMR treatments, seed treatments, and plant age determined by post hoc analyses using Tukey’s test (P < 0.05).

Total average mass gain (%) was calculated from the mean of each caterpillar’s daily growth. Mass gain analysis for the effect of BMR (ANOVA: P = 0.0088; Fig. 3a) on caterpillars fed on fresh leaves continuously, found that caterpillars fed on BMR plants (43.11 ± 8.00) gained significantly more mass than caterpillars on the artificial diet control (8.85 ± 2.79, P = 0.0065), yet for both there was no significant difference with mass gain on non-BMR plants (26.89 ± 3.88). Mass gain analysis for the effect of seed treatment (ANOVA: P = 0.0066, Fig. 3b) on caterpillars fed on fresh leaves found that caterpillars fed on untreated plants (45.5 ± 9.69) gained significantly more mass than caterpillars on artificial diet control (8.85 ± 2.79; P = 0.046), yet for both there was no significant difference with mass gain on Treated plants (30.18 ± 4.07). Mass gain analysis for the effect of plant age (ANOVA: P < 0.001, Fig. 3c) on caterpillars fed on fresh leaves found that caterpillars fed on 10-day old plants (57.63 ± 11.1) gained significantly more mass (ANOVA: P < 0.001, Fig. 3c) than those fed on 25 (20.86 ± 5.76; P = 0.0053), and 60-day old plants (14.06 ± 1.12; P < 0.0001).

#### Time to pupation

**Figure 4:**
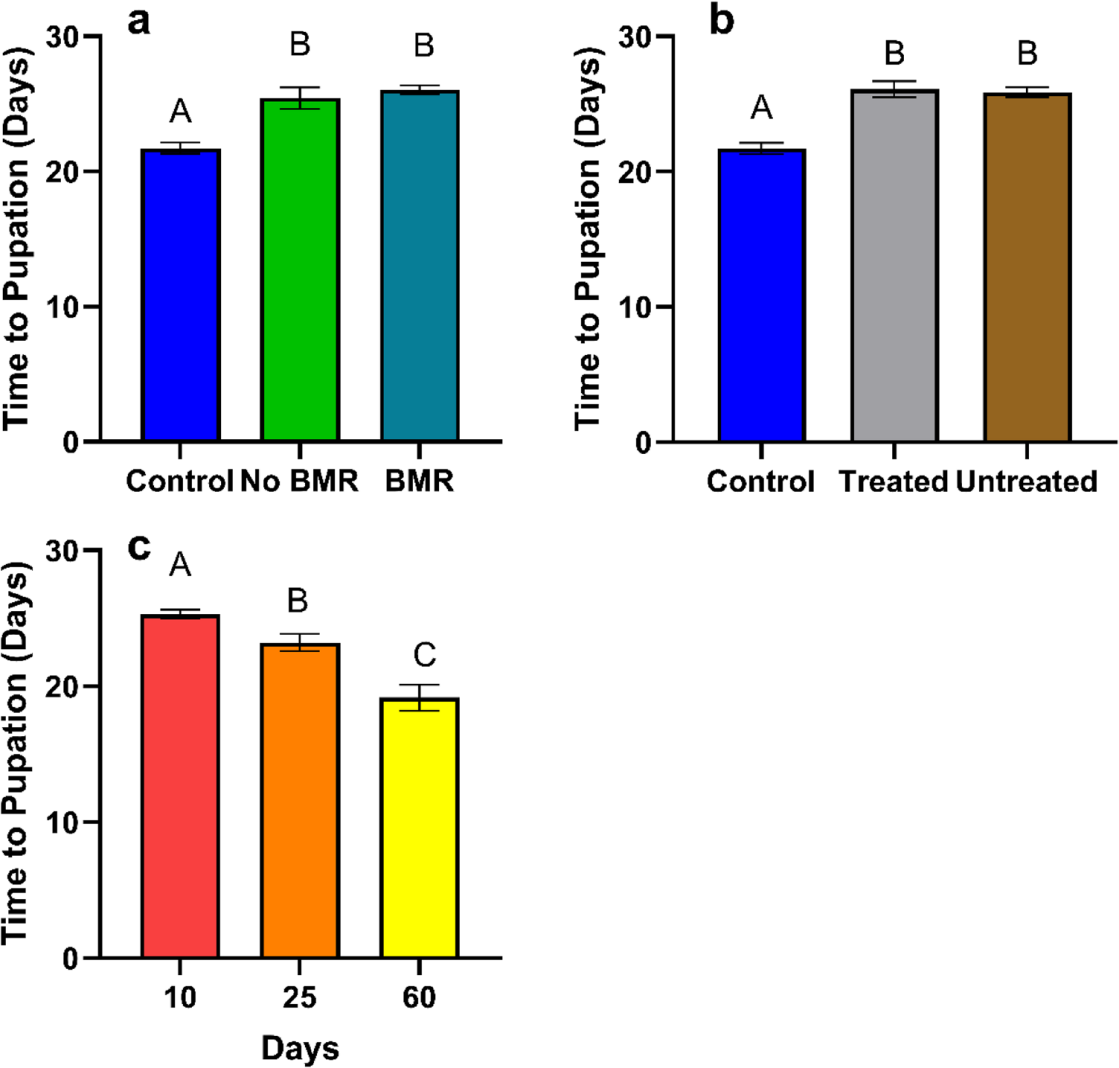
Mean time to pupation of continuous leaf exposure experiments for caterpillars on (**a**) Artificial diet control, non-BMR, BMR. (**b**) Artificial diet control, seed-treated, no seed treatment (**c**) 10-day old plants, 25-day old plants, and 60-day old plants. Each error bar is constructed using 1 standard error from the mean, different letters above the bars indicate significant differences among time to pupation between BMR treatments, seed treatments, and plant age determined by post hoc analyses using Tukey’s test (P < 0.05).

Time to pupation was calculated from how long it took caterpillars to pupate from their hatching date. Time to pupation (days) analysis for the effect of BMR presence (ANOVA: P < 0.0001, Fig. 4a) on pupation times found no significant difference in pupation times between caterpillars on BMR leaves (26.02 ± 0.31) and non-BMR leaves (25.4 ± 0.99), yet BMR (P < 0.0001) and non-BMR (P = 0.0003) both took significantly longer to pupate than the artificial diet control (21.72 ± 0.46). Time to pupation analysis for the effect of seed treatments (ANOVA: P < 0.0001, Fig. 4b) on pupation times found no significant difference in pupation times between caterpillars on treated leaves (26.1 ± 0.65) and untreated leaves (25.9 ± 0.34) yet treated (P < 0.0001) and untreated (P < 0.0001) both took significantly longer to pupate than the artificial diet control (21.72 ± 0.46). Time to pupation analysis for the effect of plant age (ANOVA: P < 0.0001, Fig. 4c) on pupation times found caterpillars fed on 10-day old leaves (25.3 ± 0.33; P = 0.0137) took significantly longer to pupate than caterpillars fed on 25-day leaves (23.2 ± 0.75; P = 0.0018), which took significantly longer to pupate than those fed on 60-day leaves (19.2 ± 0.98).

#### Pupal mass

**Figure 5:**
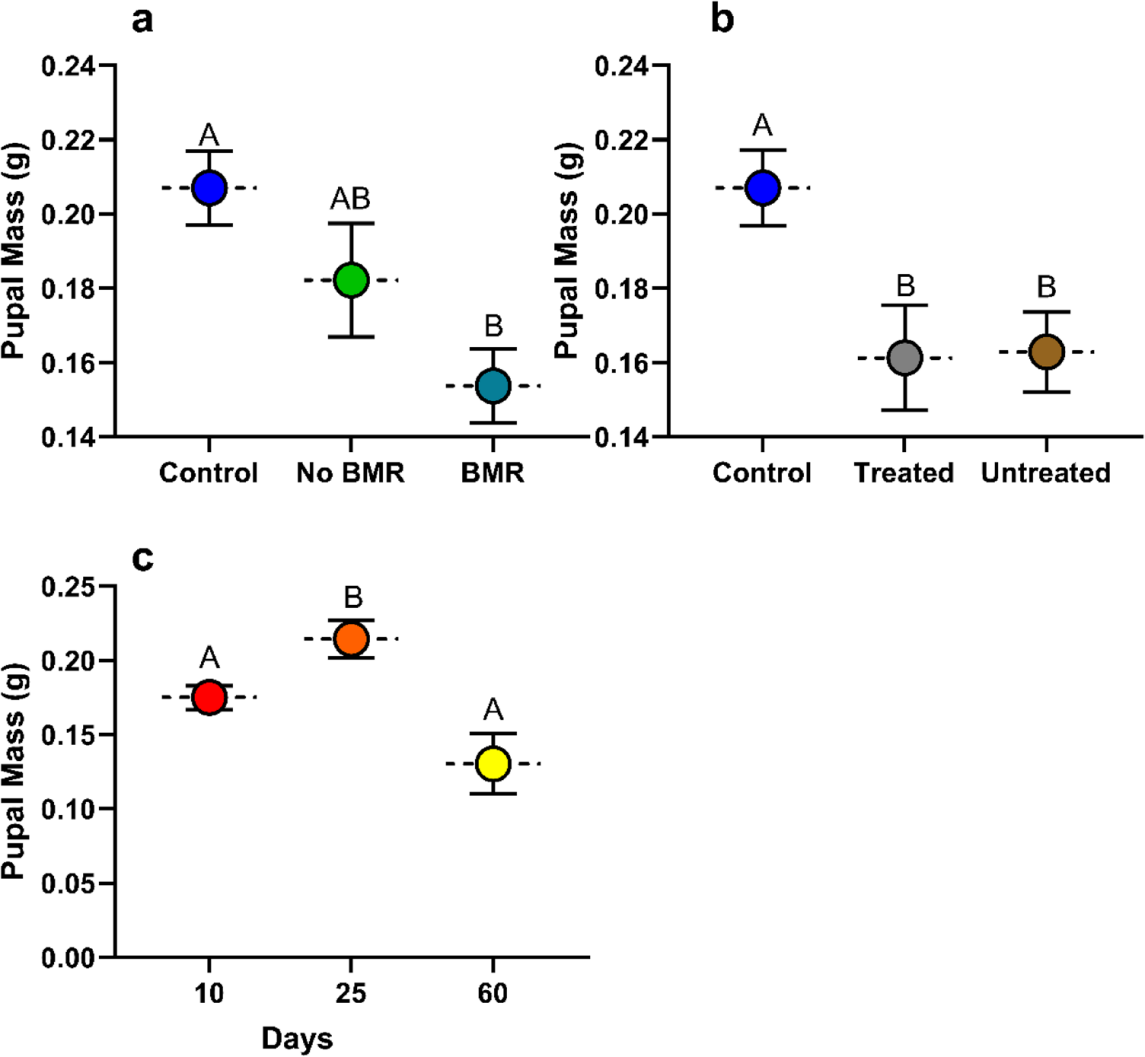
Mean pupal mass of continuous leaf exposure experiments for caterpillars on (**a**) Artificial diet control, non-BMR, BMR. (**b**) Artificial diet control, seed-treated, no seed treatment. (**c**) 10-day old plants, 25-day old plants, and 60-day old plants. Each error bar is constructed using 1 standard error from the mean, different letters above the bars indicate significant differences among pupal mass between BMR treatments, seed treatments, and plant age determined by post hoc analyses using Tukey’s test (P < 0.05).

Pupal mass was calculated from pupal mass after the first day of pupation. Pupal mass analysis for the effect of BMR presence (ANOVA: P = 0.0019, Fig. 5a) on pupal mass found that caterpillars that pupated from feeding on BMR leaves (0.154 ± 0.005) had significantly lower pupal mass than the artificial diet control (0.207 ± 0.013; P = 0.0012), although neither had a significant difference in pupal mass than caterpillars fed on non-BMR leaves (0.182 ± 0.012). Pupal mass analysis for the effect of seed treatment (ANOVA: P = 0.0063, Fig 5b) on pupal mass found no significant difference between caterpillars fed on treated leaves (0.161 ± 0.007) and untreated leaves (0.163 ± 0.007), yet the artificial diet control pupae (0.207 ± 0.013) were significantly heavier than both (P=0.0305; P=0.0123 respectively). Pupal analysis for the effect of plant age (ANOVA: P = 0.0026, Fig. 5c) on pupal mass found that caterpillars that pupated from feeding on 25-day old plants (0.214 ± 0.016) were significantly heavier than those fed 10-day old plants (0.175 ± 0.042; P = 0.0311), and 60-day old plants (0.130 ± 0.033; P = 0.0031).

#### Adult mass

**Figure 6:**
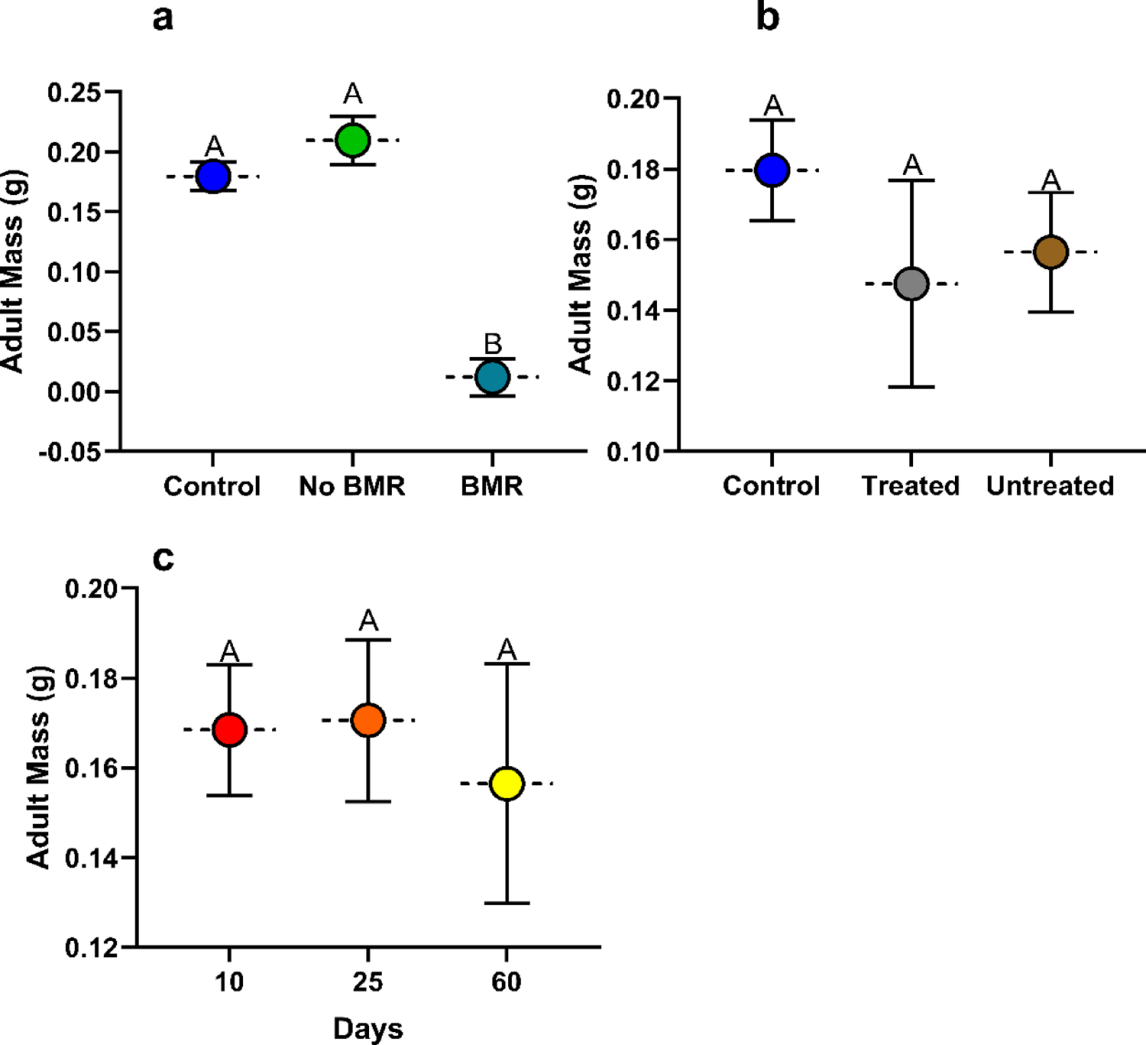
Mean adult mass of Continuous leaf exposure experiments for caterpillars on (**a**) Artificial diet control, non-BMR, BMR. (**b**) Artificial diet control, seed -treated, no seed treatment. (**c**) 10-day old plants, 25-day old plants, and 60-day old plants. Each error bar is constructed using 1 standard error from the mean, different letters above the bars indicate significant differences among adult mass between BMR treatments, seed treatments, and plant age determined by post hoc analyses using Tukey’s test (P < 0).

Adult mass was calculated from eclosed pupae. Adult mass analysis for the effect of BMR presence (ANOVA: P = 0.0030, Fig. 6a) on adult mass found that FAW that had fed on BMR leaves (0.179 ± 0.051) were significantly lower in mass than those fed on non-BMR leaves (0.209 ± 0.024; P = 0.0044), and the artificial diet control (0.17 ± 0.012; P = 0.0152). Adult mass analysis for the effect of seed treatment (ANOVA: P = 0.4509, Fig. 6b) on adult mass found that there was no significant difference between adults that had fed on treated (0.148 ± 0.032) leaves, untreated (0.156 ± 0.019) leaves, and the artificial diet control (0.179 ± 0.012). Adult mass analysis for the effect of plant age (ANOVA: P = 0.9031, Fig. 6c) on adult mass found that there was no significant difference between adults that had fed on 10 (0.168 ± 0.013), 25 (0.170 ± 0.02), and 60-day old plants (0.156 ± 0.028).

#### Plant-based diet exposure experiment

##### Total average mass gain

**Figure 7:**
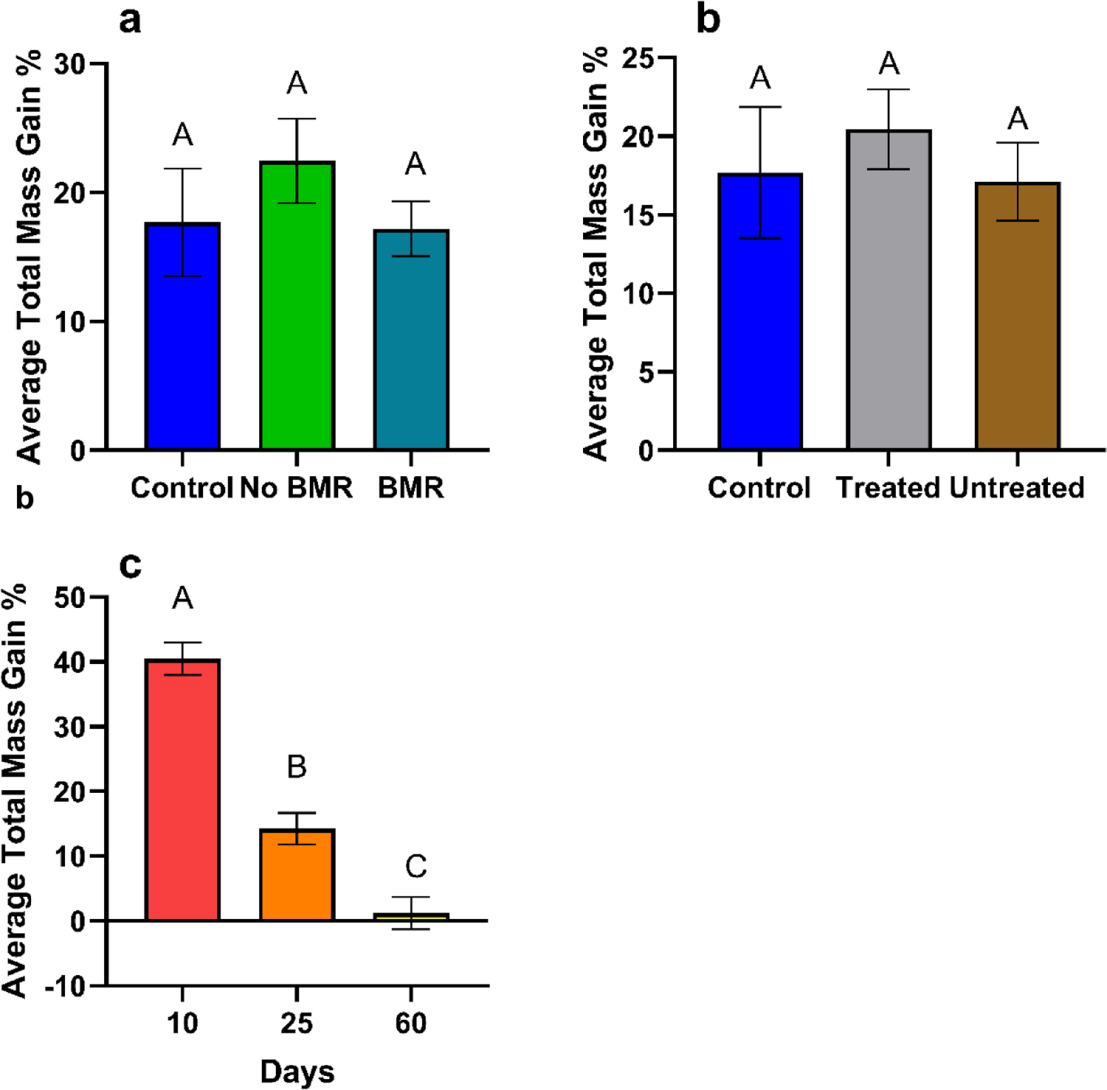
Mean total average mass gain of plant-based artificial diet experiments for caterpillars on (**a**) Artificial diet control, non-BMR, BMR. (**b**) Artificial diet control, seed treated, no seed treatment. (**c**) 10-day old plants, 25-day old plants, and 60-day old plants. Each error bar is constructed using 1 standard error from the mean, different letters above the bars indicate significant differences among total mass gain between BMR treatments, seed treatments, and plant age determined by post hoc analyses using Tukey’s test (P < 0.05).

Total average mass gain was calculated from the mean of each caterpillar’s daily growth. Mass gain analysis for the effect of BMR presence (ANOVA: P = 0.3854, Fig. 7a) on caterpillars fed on different artificial leaf-diets found no significant differences in mass gain between BMR diets (17.17 ± 2.20), non-BMR diets (22.4 ± 3.57), and the artificial diet control (17.6 ± 2.28). Mass gain analysis for the effect of seed treatment (ANOVA: P = 0.3854, Fig. 7b) on caterpillars fed on seed treated artificial leaf-diets found no significant differences (ANOVA: P = 0.3854, Fig. 7b) in mass gain between treated diets (20.5 ± 0.272), untreated diets (17.1 ± 2.60), and the artificial diet control (17.6 ± 2.28). Mass gain analysis for the effect of plant age (ANOVA: P < 0.0001, Fig. 7c) on caterpillars fed on artificial leaf-diets from different aged plants found caterpillars gained significantly more mass (P < 0.0001) on 10-day old plants (40.5 ± 3.95) than 25-day old plants (14.2 ± 1.37), which were significantly heavier than those on 60-day old plants (1.12 ± 1.02; P = 0.0005).

##### Time to pupation

**Figure 8:**
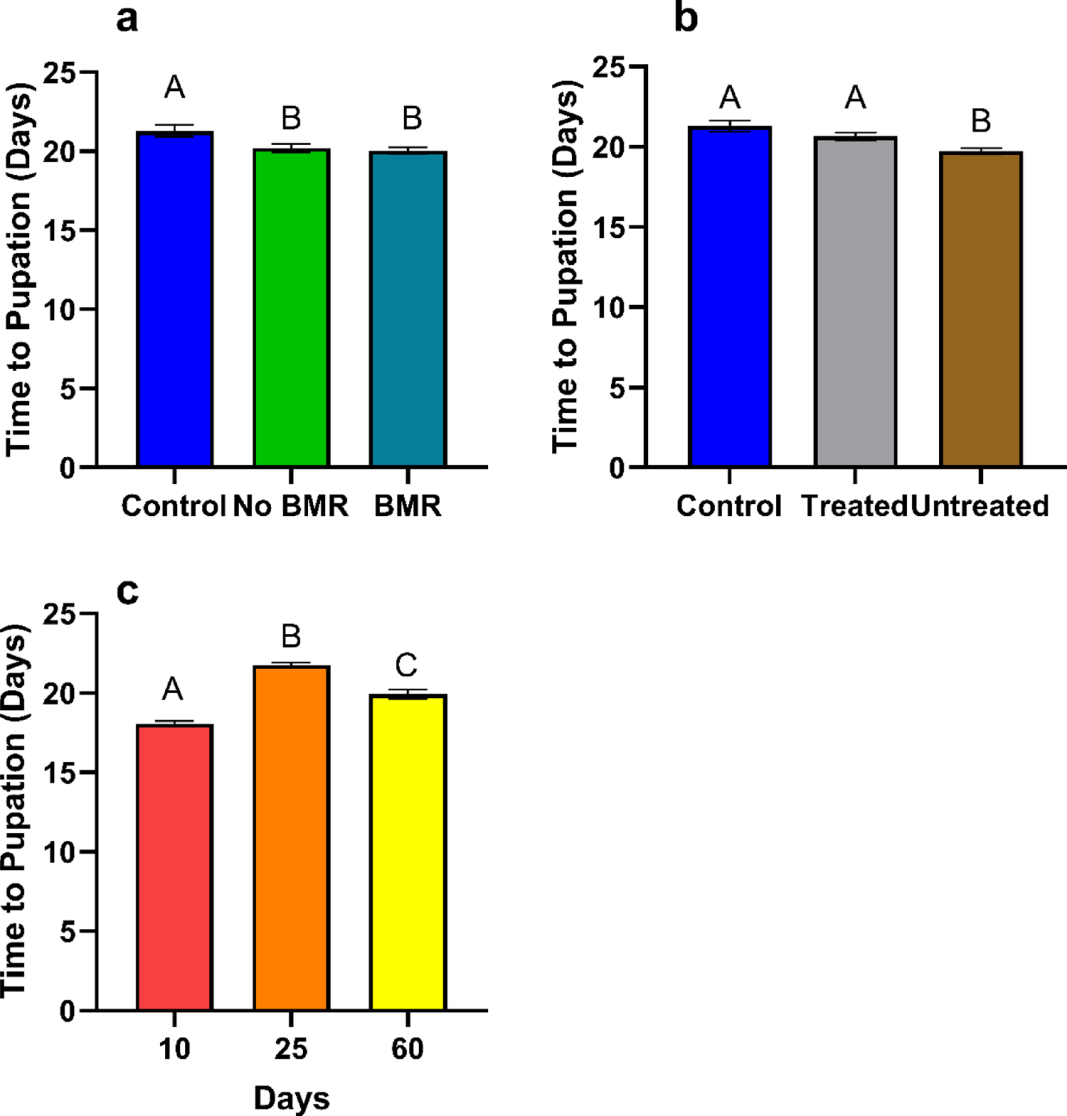
Mean time to pupation of plant-based artificial diet experiments for caterpillars on (**a**) Artificial diet control, non-BMR, BMR (**b**) Artificial diet control, seed-treated, no seed treatment (**c**) 10-day old plants, 25-day old plants, and 60-day old plants. Each error bar is constructed using 1 standard error from the mean, different letters above the bars indicate significant differences among time to pupation between BMR treatments, seed treatments, and plant age determined by post hoc analyses using Tukey’s Test (P < 0.05).

Time to pupation was calculated from the length of time required from hatching to pupation. Time to pupation analysis for the effect of BMR presence (ANOVA: P = 0.0134, Fig. 8a) on pupation times found no significant difference in pupation times between caterpillars on BMR leaf-diets (20.05 ± 0.202) and non-BMR leaf diets (20.2 ± 0.238), yet BMR (P = 0.0102) and non-BMR (P = 0.0471) both took significantly less time to pupate than the artificial diet control (21.29 ± 0.45). Time to pupation analysis for the effect of seed treatment (ANOVA: P = 0.0003, Fig. 8b) on pupation times found significantly faster pupation times (P = 0.0120) between caterpillars on untreated leaf-diets (19.7 ± 0.177) than treated leaf-diets (20.6 ± 0.265) and the artificial control diet (25.9 ± 0.34; P = 0.0007). Time to pupation analysis for the effect of plant age (ANOVA: P < 0.0001, Fig. 8c) on pupation times found caterpillars fed on 10-day old diets (18.05 ± 0.088) were significantly faster (P < 0.0001) than 60-day old diets (19.93 ± 0.267), which were significantly faster (P < 0.0001) than those fed on 25-day old diets (21.75 ± 0.172).

##### Pupal mass

**Figure 9:**
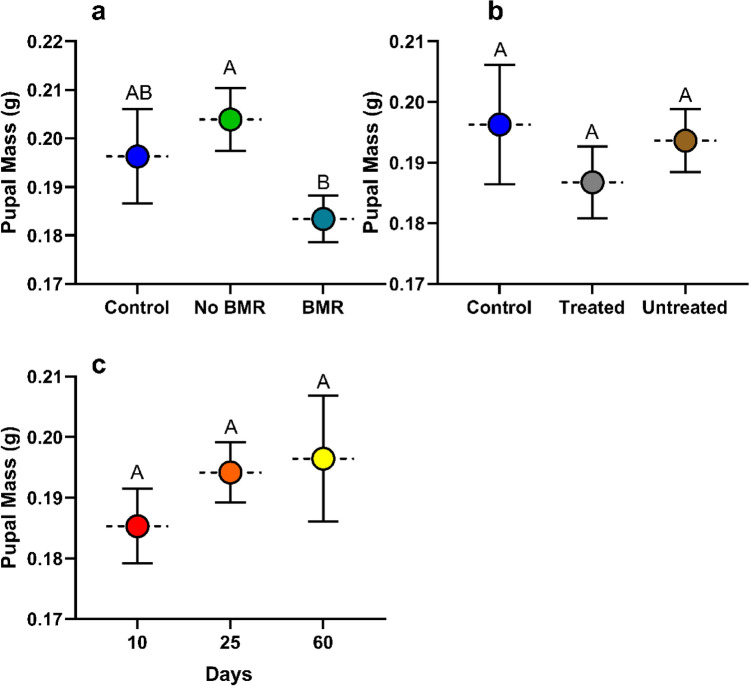
Mean pupal mass of plant-based artificial diet experiments for caterpillars on (**a**) Artificial diet control, non-BMR, BMR. (**b**) Artificial diet control, seed-treated, seed treatment. (**c**) 10-day old plants, 25-day old plants, and 60-day old plants. Each error bar is constructed using 1 standard error from the mean, different letters above the bars indicate significant differences among pupal mass between BMR treatments, seed treatments, and plant age determined by post hoc analyses using Tukey’s test (P < 0.05).

Pupal mass was calculated from pupal mass after the first day of pupation. Pupal mass (g) analysis for the effect of BMR presence (ANOVA: P = 0.0358, Fig. 9a) on pupal mass found that caterpillars that pupated from feeding on BMR diets (0.183 ± 0.004) were significantly lower (P = 0.0311) in mass than those on non-BMR diets (0.204 ± 0.008), although neither were significantly different from the artificial diet control (0.196±0.10). Pupal mass analysis for the effect of seed treatment (ANOVA: P = 0.5938, Fig. 9b) on pupal mass found no significant difference in mass between caterpillars that pupated by feeding on treated leaf-diets (0.187 ± 0.007), untreated leaf-diets (0.194 ± 0.004), and the artificial diet control (0.196 ± 0.057). Pupal mass analysis for the effect of plant age (ANOVA: P= 0.4662, Fig. 9c) on pupal mass found no significant difference in mass between caterpillars that pupated on 10 (0.185 ± 0.006), 25 (0.194 ± 0.005), and 60-day old leaf-diets (0.196 ± 0.0102).

##### Adult mass

**Figure 10:**
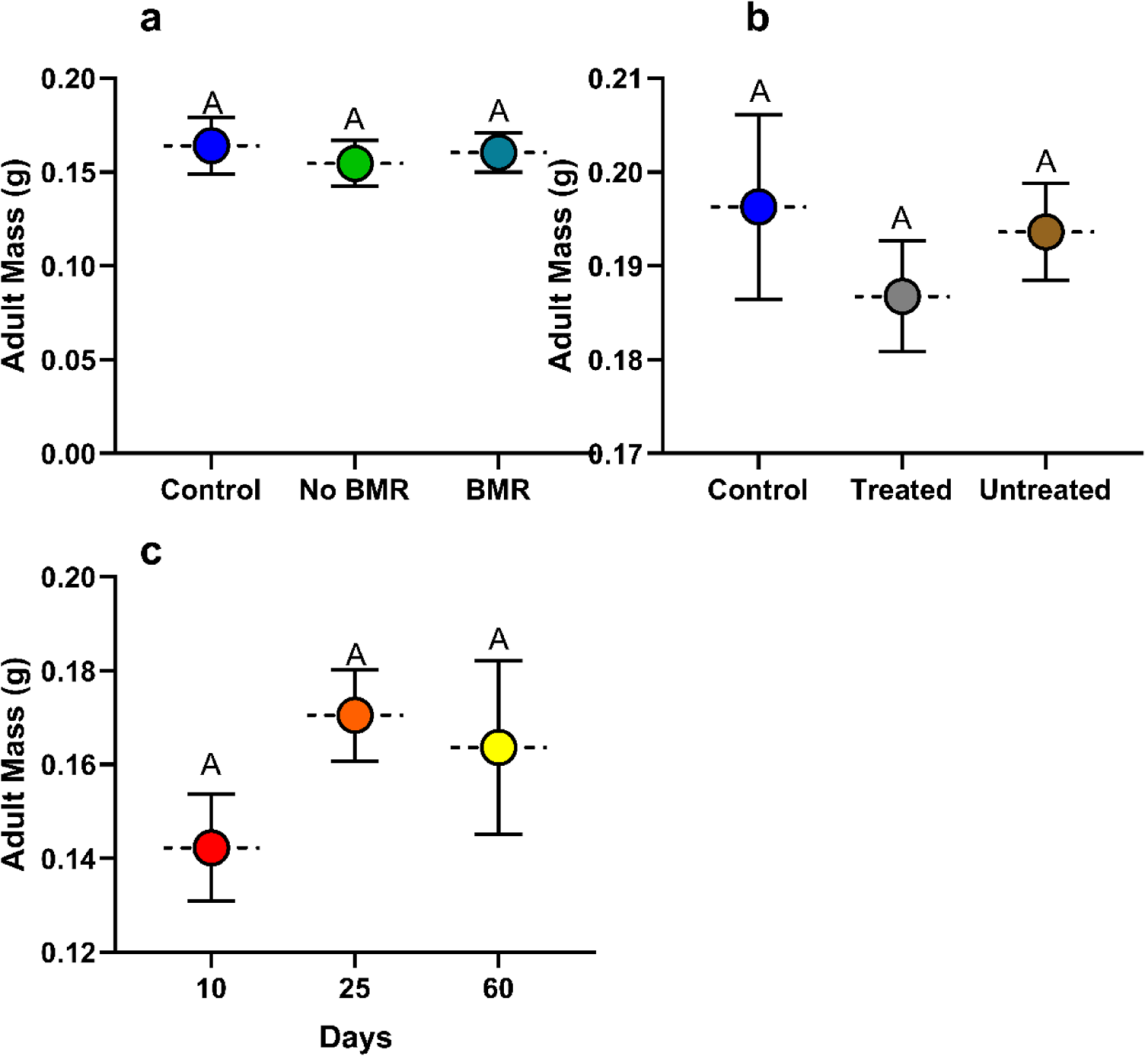
Mean adult mass of plant-based artificial diet experiments for caterpillars on (**a**) Artificial diet control, non-BMR, BMR. (**b**) Artificial diet control, seed treated, no seed treatment. (**c**) 10-day old plants, 25-day old plants, and 60-day old plants. Each error bar is constructed using 1 standard error from the mean, different letters above the bars indicated significant differences among adult mass between BMR treatments, seed treatments, and plant age determined by post hoc analyses using Tukey’s test (P < 0.05).

Adult mass was calculated from eclosed pupae. Adult mass analysis for the effect of BMR presence (ANOVA: P = 0.8786, Fig. 10a) in leaf-diets on adult mass found no significant difference in adult mass on BMR diets (0.160±0.012), non-BMR diets (0.155 ± 0.012), and the artificial diet control (0.164 ± 0.010). Adult mass analysis for the effect of seed treatment leaf-diets (ANOVA: P = 0.7736, Fig. 10b) on adult mass found no significant difference in adult mass between treated diets (0.166 ± 0.02), untreated diets (0.155 ± 0.008), and the artificial diet control (0.164±0.0544). Adult mass analysis for the effect of plant age (ANOVA: P = 0.1682, Fig. 10c) on adult mass found no significant differences in adult mass between adults who fed as larvae on 10 (0.142 ± 0.007), 25 (0.170 ± 0.011), and 60-day old plant diets (0.164 ± 0.025).

## Discussion

In this study, we evaluated the effect of BMR, seed treatment, and plant age on the growth and development of FAW. We monitored BMR sorghum-sudangrass x FAW interactions through multiple lenses: 48-hour snapshot exposure, continuous leaf feeding, and life cycle on artificial diet fortified with fresh leaf extract. Our hypothesis that seed treatments would not have an effect on FAW growth and development, or on their ability to damage sorghum-sudangrass was supported, with no effects from seed treatment. However, we also hypothesized that older plants would be the most detrimental to FAW, yet while that was mostly supported, in the 48 h exposure snapshot experiment we saw the opposite. As for BMR, we hypothesized differential effects throughout every experiment; however, BMR had no effect on FAW in larval stages until the adult and pupal stages.

The snapshot exposure experiments allowed us to understand the immediate response of FAW larvae to the seed treatment, to BMR, but also how plant age affects these larvae in the short term. When sensing herbivory, plants are able to respond by mobilizing local defenses to areas of herbivore feeding, or by releasing volatile organic compounds with the purpose of attracting natural enemies^47,48^. FAW, however, are highly mobile and have been shown to evade plant defenses by damaging a plant and moving on to another plant, or by feeding on different tissues of the same plant^49–51^. The snapshot exposure experiments give insight into how FAW are affected when spatially constrained and unable to perform movement-based evasion of plant defenses. Both continuous experiments exploring the entire FAW life cycle shed light on if FAW can overcome adverse effects in their diet later in their life cycle, or if detriments brought upon by our factors are insurmountable. While some studies have observed the effect of one of these factors, very few studies have taken them together and thoroughly monitored the FAW growth and development.

First, we found that seed treatments appeared to have almost no effect on FAW growth and development. These findings echo those of Muraro’s group^22^ which found no effect from seed treatments on FAW after 20 days of sowing, alongside those of Assefa^52^ which found seed treatments as ineffective and inconclusive for FAW control in Ethiopia. These results are also in line with Thrash’s group^23^, in which they found FAW survival to be the most affected by seed treatments. The seed treatment in our experiment, spinosad, acts on the ganglia and central nervous system (CNS) of target insect pests and disrupting acetylcholine pathways and receptors^53,54^. Spinosad is biologically derived from soil bacteria, *Saccharopolyspora spinosa*, and has been reported to be promising for integrated pest management because of its biological origin, low off-target effects^55^. Additionally, it is not known to share cross-resistance with more widely used insecticides^55^. However, the issue our study and more recent research has shown is that spinosad is not always effective, and almost always requires concurrent use of another insecticide to achieve efficient control, or a dramatic increase in dosage, as 200 ppm showed little effects while 1000–2000 ppm displayed high mortality in Lepidoptera^56,57^. While spinosad has been shown to be effective in controlled tests, Hertlein’s group^57^ also found that full sunlight and a large presence of organic matter may reduce spinosad’s efficacy, which may explain why we saw no effect of the insecticide in our snapshot experiment performed in a greenhouse, regardless of the phonological stage of the plant. Regarding seed treatments, our results indicate that seed treatments other than spinosad should be further evaluated for FAW control, alongside novel pest management strategies.

Plant age as a factor in FAW growth and development is highly important as it serves to develop action and economic thresholds^58,59^. Predicting pest infestations, and specifically FAW infestations, has been a prime concern for researchers and growers, and understanding what plant stages are most resistant or susceptible is key to developing management practices based around planting dates with expected pest infestation periods, and applying this data to changing pest patterns alongside global warming^60–63^. Our most conclusive results were found when monitoring the effect of plant age on FAW. We found that FAW exposed to 10-day old sorghum-sudangrass plants for a short period, i.e. 48 h exposure, gained less mass after this period, had the most herbivory damage, and gained the most mass in both continuous leaves and diet experiments; however, on continuous leaf diet they took the longest to pupate. This contradicts our other pupation time results as they were found to pupate the fastest on plant-based artificial diets. FAW on continuous leaf diets had the lowest growth on 60-day old plants, alongside losing significantly more mass in the final days of development leading to pupation on plant-based diets when compared to those feeding on 10-day old diets. One study^64^ that analyzed sorghum waxes found that different stages in plant growth had different amounts of wax, and different chemical compositions of wax, both of which were highly variable until the grain heads became apparent. Although FAW did not pupate slower than those on 25-day old diets (the slowest pupation rate), they pupated significantly slower than those on 10-day old diets. While these adverse effects of 25-day old plants are not uncommon, they are usually seen in the reproductive stages of the plant, while in sorghum this is close to the transition point between vegetative and reproductive stages^65–67^.

A major driver of this study was the lack of a body of work on BMR as a host plant resistance trait, and therefore more work on BMR may shed light on these promising results and their mechanism(s). The effect of FAW on sorghum-sudangrass and sorghum has been well studied, especially with regard to FAW feeding leading to elevated levels of flavonoid compounds in sorghum-FAW interactions^48^. Flavonoids are crucial in providing host plant resistance^46,68^, and in sorghum, this being further supported by a sequential herbivory study by Kundu^69^ in which FAW feeding first lead to drastic defense trait induction, measured by accumulation of total flavonoids. Sorghum flavonoids also have been shown to impose stress on another feeding guild insect, corn leaf aphids (*Rhopalosiphum maidis*), increasing the likelihood of alate development and reduction in colonization numbers^70^. Our knowledge gap therefore lies in understanding how BMR affects these defenses. Our results indicate that that when fed on BMR fresh leaves continuously, FAW had significantly lower pupal mass than the artificial diet control and a significantly lower adult mass than both the artificial diet control and non BMR plants. In the same vein, we found that when fed on plant-based artificial diets, FAW that fed on BMR diets also had significantly lower pupal mass than their non BMR counterparts. In Lepidoptera, pupal mass is a traditional indicator of adult traits, which translate into fitness, therefore reductions in pupal mass have been shown to hinder the overall fitness of the insect^71,72^. This data supports the idea that BMR does have an effect on herbivore performance and shows that further studies into the effect of BMR on pest insects should be expanded outside of FAW. Also, since this was consistent across ages of the plant and seed treatments, we predict that BMR is affecting FAW possibly through sorghum defense pathways. One possibility is through the modification of lignin biosynthesis in BMR mutants that leads to cascading effects in the plant’s ability to respond to biotic factors, primarily herbivore pressure^73^. One such hypothesis is that the reduced lignin pathway leads to an increase in flavonoid synthesis, which are known for their defense properties^74^. While it is understood that there are tradeoffs due to BMR mutations within the plant, such as changes in metabolites, silencing, or overexpression of genes, further studies are required to understand the effect of BMR on surface defenses, primarily epicuticular waxes and trichomes, which has not been studied thoroughly^48,69,75^.

The adverse effects of BMR and later plant ages (25 and 60 days) on FAW growth and development found in this study also warrant deeper examination into their mechanism and modes of action. For example, plant growth stage being harmful to FAW in later stages is most likely due to altered wax concentrations^76,77^ and secondary metabolites^78,79^ found on older leaves. However, this does not explain the few cases where 25-day old leaves were more harmful to FAW growth and development than 60-day old leaves, although this could be tested by quantifying the wax content and abundance since only few studies have examined it^80^.

To conclude, our data from snapshot exposure, continuous fresh leaf exposure, and plant-based artificial diets- a collective and comprehensive examination of FAW growth and development, strongly supports the idea that BMR and plant age play a large role in deterring FAW herbivory by impeding their growth and development. Moving forward, the mechanisms of how these coalesce with plant resistance should be explored through studies that quantify differences in primary and secondary metabolites, and direct and indirect defenses, an area we are currently exploring.

## Data Availability

The datasets generated and analyzed during the current study are available from the corresponding author on reasonable request.
